# A Pharmacogenetic Study of VDR fok1 and TYMS Polymorphisms and Their Association With Glucocorticoid-Induced Osteonecrosis in Egyptian Children With Acute Lymphoblastic Leukemia

**DOI:** 10.3389/fonc.2018.00541

**Published:** 2018-11-23

**Authors:** Dina ElHarouni, Dina Yassin, Nesreen Ali, Seham Gohar, Iman Zaky, Hassan Adwan, Iman Sidhom

**Affiliations:** ^1^Pharmacology and Toxicology Department, Faculty of Pharmacy and Biotechnology German University in Cairo, New Cairo, Egypt; ^2^Clinical Pathology Department, Children Cancer Hospital Egypt and National Cancer Institute Cairo University, Cairo, Egypt; ^3^Pediatric Oncology Department, Children Cancer Hospital Egypt and National Cancer Institute Cairo University, Cairo, Egypt; ^4^Pediatric Oncology Department Children Cancer Hospital Egypt, Cairo, Egypt; ^5^Radiology Department, Children Cancer Hospital Egypt and National Cancer Institute Cairo University, Cairo, Egypt

**Keywords:** VDR, Vit D receptor, TYMS polymorphism, glucocorticoid, osteonecrosis, ALL – acute lymphoblastic leukemia

## Abstract

**Purpose:** Osteonecrosis is a significant toxicity resulting from the treatment of pediatric Acute Lymphoblastic Leukemia (ALL). This study aimed to investigate the relationship between vitamin D receptor fok1 (VDR fok1) and thymidylate synthase (TYMS) gene polymorphisms with the glucocorticoid (GC) induced osteonecrosis (ON) in Egyptian pediatric ALL patients. In addition, to identify the possible association of genetic polymorphisms with other factors such as gender and ALL subtypes.

**Patients and Methods:** A retrospective case-control study was conducted on 102 pediatric ALL patients under the age of 18 who were treated at Children Cancer Hospital Egypt according to St Jude SR/HR total XV protocol. The recruited patients were composed of 51 cases who developed GC-induced osteonecrosis and 51 age- and gender-matched patients who received glucocorticoids but remained osteonecrosis-free (controls). Genotyping of the VDR fok1 and TYMS genes was performed using restriction fragment length polymorphism (RFLP) and conventional PCR, respectively.

**Results:** For the total 102 studied patients, the VDR fok1 single nucleotide polymorphisms (SNPs) frequency distribution were TT (8.8%), CT (34.3%), and CC (56.9%), while the TYMS tandem repeat gene variations were reported as 2R2R (20.6%), 2R3R (45.1%), and 3R3R (34.3%). VDR fok1 and TYMS polymorphic variants showed no association neither with gender; *P*-values 0.3808 and 0.1503, respectively, nor with ALL subtypes; *P*-values 0.9396 and 0.6596, respectively. The VDR fok1 polymorphisms showed a significant association with the development of ON; *P*-value = 0.003, on the other hand, TYMS tandem repeats did not show significant impact on osteonecrosis development; *P*-value = 0.411.

**Conclusion:** This study showed a significant association between the VDR fok1 polymorphism and osteonecrosis. Such clinical pharmacogenetics results would be promising to discuss the possibility of dose adjustments aiming a regimen with the highest efficacy and least toxicity.

## Introduction

Acute Lymphoblastic Leukemia (ALL) is the most prevalent cancer type in children (1). Progresses in the therapeutic management of pediatric ALL have resulted in increasing the cure rates up to 90% (2). The combination of methotrexate, 6-mercaptopurine and glucocorticoids constitute the standard backbone of ALL regimens. However, significant toxicities remain a major risk factor that causes long-term morbidity and decreased quality of life (3).

Glucocorticoid (GC)-induced-Osteonecrosis (ON) is a challenging complication encountered during modern chemotherapy for childhood ALL. Osteonecrosis also known as Avascular Necrosis (AVN) has been increasingly reported in pediatric ALL and presents a challenging complication (3). It is a disabling clinical disease characterized by a decrease in osteoblastic activity and increased bone resorption (2). Morbidity is caused by progressive joint damage which may lead to total joint replacement. GC-induced-ON is considered a multifactorial disease resulting from clinical risk factors as age, gender, and race as well as genetic factors (4). Several research investigations have demonstrated that genetic polymorphisms in drug-targeted genes are highly associated with inter-individual differences in the efficacy and toxicity of the regimen.

The Vitamin D Receptor (VDR) gene encoding for the nuclear hormone receptor for vitamin D has a significant role in calcium homeostasis. It plays a crucial role within several drug pathways, their treatment response, and toxicity, and also regulates CYP3A4 which is a key role player in drug metabolism (5). Four Single Nucleotide Polymorphisms (SNPs) of the VDR gene are significantly important in pharmacogenetic studies namely known as BsmI (rs1544410), ApaI (rs7975232), TaqI (T > C; rs731236), and FokI (C > T; rs2228570) (6).

Thymidylate Synthase (TYMS) is a key enzyme of folate metabolism and a target for several chemotherapeutic drugs such as methotrexate (7, 8). The TYMS gene has a common length polymorphism characterized by the presence of 28-base pair tandem repeats (TR) in the 5′-untranslated region (5′-UTR). There are three predominant genotypes associated with the TYMS gene: (1) homozygous with two TR (2R2R); (2) homozygous with three TR (3R3R); and (3) heterozygous with both alleles (2R3R). Increased TYMS mRNA expression and enzyme activity are associated with a greater number of tandem repeats, in turn; lower expression levels of TYMS may reduce the enzyme activity (9, 10). Therefore, the TYMS expression variability between tandem repeats may affect the folate pharmacodynamics, hence altering the lipid drugs metabolism.

Since genetic polymorphisms vary among populations, and the possible risk factors for GC-induced-ON has not been studied in the Egyptian population, therefore, we performed this study to assess the frequency of pharmacogenetic variants of VDR fok1 and TYMS genes among the studied Egyptian pediatric ALL patients and to explore their association with the occurrence of osteonecrosis.

## Patients and methods

### Patients

A case-control pharmacogenetics study was conducted on 102 pediatric ALL patients treated at Children Cancer Hospital Egypt (CCHE- 57357 Hospital). The recruited patients were composed of two groups; 51 cases who developed GC-induced-ON and 51 controls, age and gender-matched patients who received the same dose of GC but remained osteonecrosis-free.

It included ALL patients under the age of 18 years who ended a two-year continuation therapy of SR/HR St Jude total XV protocol and performed MRI of hips and Knees during therapy and at the end of treatment.

The study was approved by the Institutional Review Board (IRB) at 57357 CCHE. A written informed consent was obtained from all patient's guardians included in the study for blood sampling and genotyping.

### Treatment regimen

The most common GCs administered to pediatric ALL patients are prednisolone and dexamethasone. According to St Jude SR/HR total XV protocol, prednisone was given at a dose of 40 mg/m^2^/day daily for 28 induction days. On the other hand, dexamethasone was administered orally in the continuation therapy which lasted for 100 weeks and in the reinduction phases of chemotherapy. During the continuation phase dexamethasone was given at a dose of 12 mg/m^2^/day for 5 continuous days every 4 weeks; however; during reinduction phases I and II it was given at a dose of 8 mg/m^2^/day for 7 continuous days during weeks 7, 9, 17, and 19.

### Detection of osteonecrosis

Patients were routinely screened for osteonecrosis at CCHE-57357 by MRI examination of the hips and knees during therapy and at the end of treatment or at any time due to symptoms.

### Genotyping

**Figure 1:**
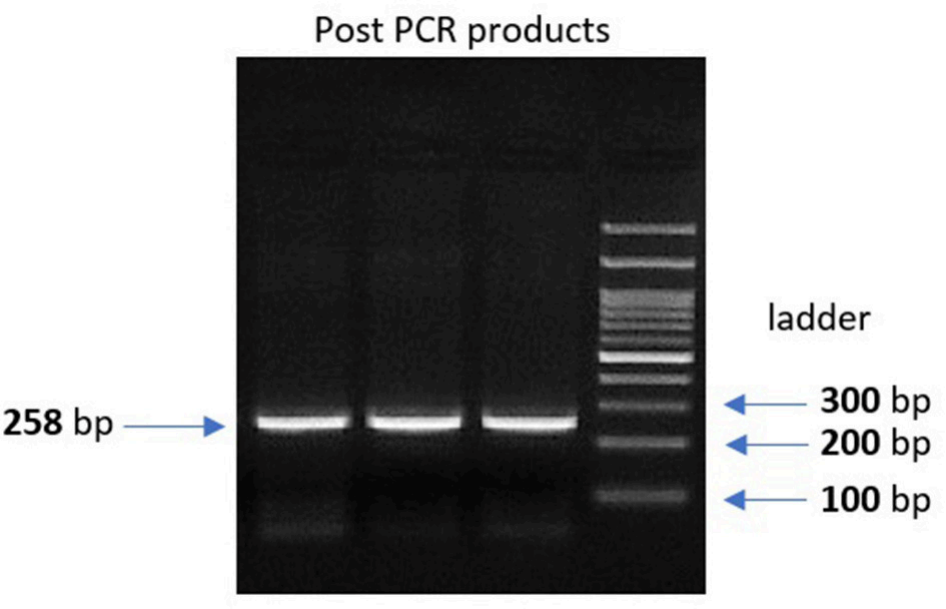
2% gel electrophoresis image for the post PCR product of VDR fok1 gene, Lanes 1, 2, 3: VDR fok1 amplification at 258 bp, Lane 4: 100 bp DNA Ladder.

**Figure 2:**
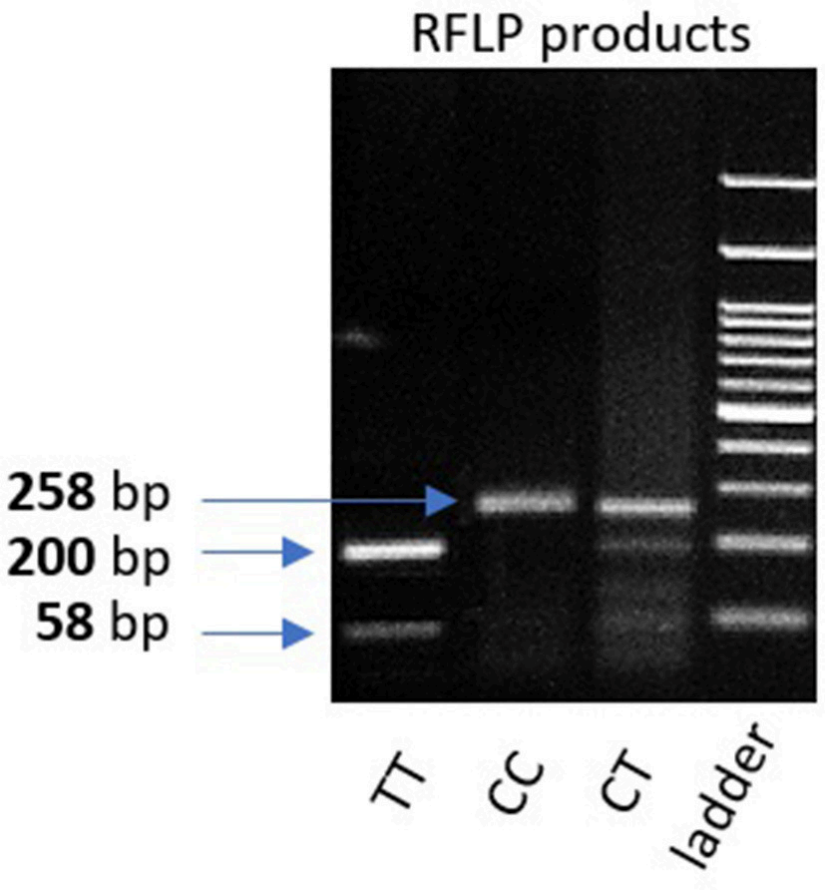
2% gel representing PCR fragments of VDR fok1 genotypes using RFLP PCR. Lane 1: The homozygous mutant type (TT) cut at one site giving 2 bands (200 and 58 bp). Lane 2: Homozygous wild type (CC) uncut, (258 bp). Lane 3: Heterozygous (CT) genotype having 3 bands (258, 200, 58 bp). Lane4: 100 bp DNA Ladder.

Genomic DNA was extracted from peripheral leukocytes of fresh blood samples using an ABIOpure Genomic DNA human Blood Extraction kit (ABIOpure™ AllianceBio, USA). Genotyping was performed for VDR fok1 SNP and TYMP 28 bp tandem repeat. The VDR fok1 *rs2228570* was amplified using 100 ng DNA sample, 2.5 μl Hot Start Taq enzyme (Soils BioDyne, USA) and 1 μl of each primer prepared with a concentration of 20 pMol/μl, the forward primer was 5′ CCCTGGCACTGACTCTGGCTCTG 3′ and the reverse primer was 5′ GAAACACCTTGCTTCTTCTCCCTCC 3′ with a total reaction volume of 50 μl. The PCR cycling conditions were 95°C for 13 min, (95°C for 30 s, 60°C for 30 s, and 72°C for 30 s) 35 cycles and a final extension at 72°C for 7 min. The amplified product was viewed on a 2% agarose gel with a band size of 258 bp as shown in Figure 1. Amplification was followed by restriction to detect the polymorphism using Restriction Fragment Length Polymorphism (RFLP) PCR using Fast digest Fok1 enzyme according to previous reports (11). Genotypes were determined by 2% agarose gel electrophoresis and defined as homozygous CC wild type uncut at 258, homozygous TT mutant type cut at 200 and 58 bp, and finally heterozygous CT at band size 258, 200, and 58 bp as shown in Figure 2.

**Figure 3:**
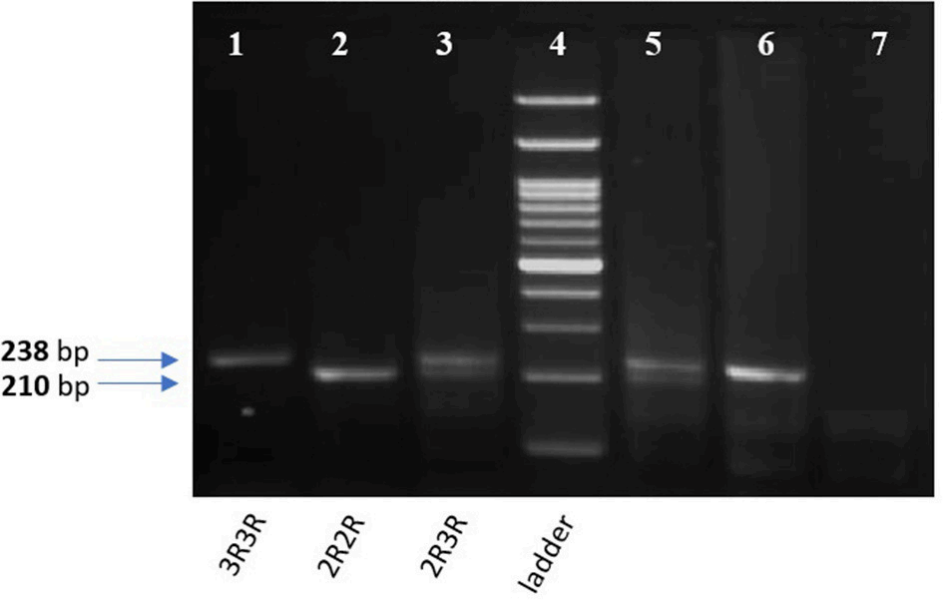
2% gel for TYMS 28bp tandem repeat PCR products. Lane 1,6: TYMS 3R homozygous repeat (238 bp). Lane 2: TYMS 2R homozygous repeat (210 bp). Lan 3,5: TYMS 2R3R heterozygous (210 and 238 bp). Lane 7: A non-template (negative control). Lane 4: 100 bp DNA Ladder.

The tandem repeat sequences in the 5′-terminal of the regulatory region of the TYMS gene was detected using conventional PCR. Genotyping was performed using 50 ng of DNA per reaction, 2 μl of regular Taq enzyme (Thermo Fisher, Germany) and 1 μl of each of the following primers with a concentration of 20 pMol/ μl: forward 5′ CGTGGCTCCTGCGTTTCC3′ and reverse 5′ GAGCCGGCCACAGGCAT 3′, the total reaction volume was 25 μl. PCR cycling conditions were: 10 min denaturation cycle at 95°C and (denaturation at 95°C for 30 s, annealing at 61°C for 45 s, and extension at 72°C for 45 s) for 35 cycles, then a final extension at 72°C for 5 min. Amplified PCR products were visualized on a 2% agarose gel with ethidium bromide. Homozygotes for the double repeat *2R2R* produced a single 210-bp band. Heterozygotes *2R3R* produced 210-bp, and 238-bp fragments and homozygotes for the triple repeat *3R3R* produced a 238-bp fragment as previously described (12), and shown in Figure 3.

### Statistical analysis

Nominal variables are presented as numbers and percentages, upon which they were compared using a Chi-Square test or Fisher's exact test. The frequencies of alleles in the pharmacogenetic analysis control group were tested against the Hardy-Weinberg equation to verify random sampling. The primary endpoint of osteonecrosis status was treated as a dichotomized variable (present vs. absent). Univariate analysis was carried out using logistic regression for each individual genotype as a predictor for osteonecrosis.

All statistical analysis was performed using the statistical programs SPSS version 15, MedCalc and Graph Pad Prism 7. To compare differences between different groups, odds ratio, and contingency 2 × 2 evaluation were used. In all statistical tests, a two-tailed *P*-value of less than 0.05 was considered statistically significant.

## Results

### Genetic polymorphism distribution frequency

For the total of 102 patients included in this study population, the VDR fok1 SNPs frequency distribution were TT (8.8%), CT (34.3%), and CC (56.9%). On the other hand, the TYMS tandem repeat gene variations were reported as 2R2R (20.6%), 2R3R (45.1%), and 3R3R (34.3%). Association of both genetic polymorphisms VDR fok1 and TYMS were tested in respect to gender (females vs. males) and ALL subtypes (B vs. T-ALL) as shown in Table 1. Among different gender subgroups, the VDR fok1 and TYMS polymorphic variants showed no association with *P*-values of 0.3808 and 0.1503 respectively. Similarly, different ALL subtypes revealed no significant difference among the VDR fok1 and TYMS genotypes, *P*-value = 0.9396 and 0.6596, respectively.

**Table 1:** Contingency analysis of VDR fok1 and TYMS tandem repeats among gender and ALL subtypes.

	**Females (*n* = 42)**	**Males (*n* = 60)**	**B-ALL**	**T-ALL**
**VDR fok1 GENOTYPE MODEL**				
TT	2 (4.8%)	7 (11.7%)	7 (9.1%)	2 (8%)
CT	17 (40.4%)	18 (30%)	27 (35.1%)	8 (32%)
CC	23 (54.8%)	35 (58.3%)	43 (55.8%)	15 (60%)
	*P* = 0.3808		*P* = 0.9396	
**TYMS TANDEM REPEATS**				
2R2R	7 (16.7%)	14 (23.3%)	16 (20.8%)	5 (20%)
2R3R	16 (38.1%)	30 (50%)	33 (42.8%)	13 (52%)
3R3R	19 (45.2%)	16 (26.7%)	28 (36.4%)	7 (28%)
	*P* = 0.1503		*P* = 0.6596	

*VDR, Vitamin D Receptor; TYMS, Thymidylate Synthase; ALL, Acute Lymphoblastic Leukemia*.

### Osteonecrosis pharmacogenetics analysis

#### VDR *fok1* genotype model

**Figure 4:**
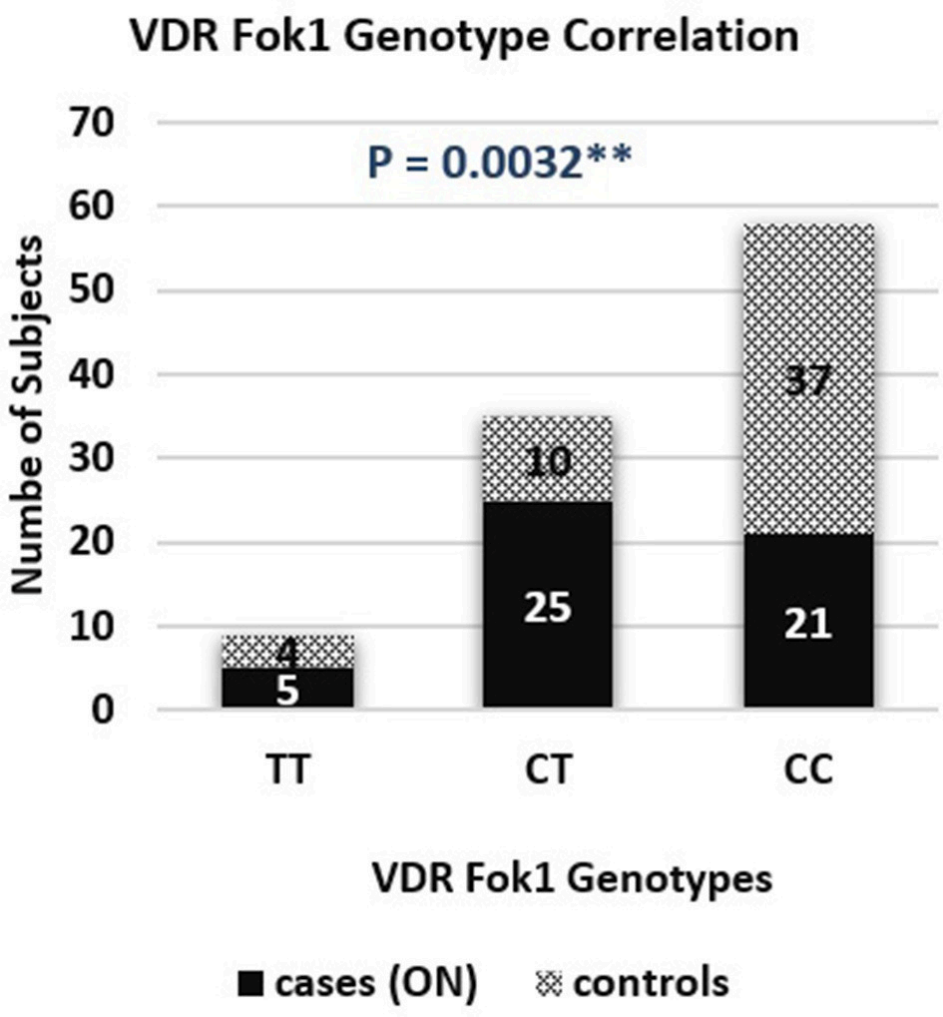
VDR fok1 genotype correlation bar chart in osteonecrosis cases and controls.

A genotype association of VDR fok1 gene was conducted in this study and revealed the following results: The homozygous mutant genotype TT was 9.8% of the ON cases while in the matched control group 7.8%. The heterozygous CT genotype was 49% in the ON cases and 19.6% in the controls. The homozygous wild type CC genotype was reported to be 41.2% and 72.6% in the ON cases and control group respectively. The VDR fok1 polymorphisms showed a significant association with the development of ON with a *P*-value of 0.003 as shown in Table 2 and Figure 4.

**Table 2:** VDR fok1 and TYMS tandem repeats in cases with osteonecrosis vs. controls (*N* = 102).

	**Osteonecrosis**	**Control**	**Odds ratio**	**95% CI**	***P*-value**
	**(*n* = 51)**	**(*n* = 51)**			
**VDR fok1 GENOTYPE MODEL**					
TT	5 (9.8%)	4 (7.8%)			
CT	25 (49%)	10 (19.6%)			0.00326
CC	21 (41.2%)	37 (72.6%)		
**VDR fok1 DOMINANT ALLELE**					
TT and CT	30 (58.8%)	14 (27.5%)	3.776	(1.646 to 8.662)	0.0025
CC	21 (41.2%)	37 (72.5%)		
**TYMS TANDEM REPEATS**					
2R2R	7 (13.7%)	14 (27.5%)		
2R3R	26 (51%)	20 (39.2%)			0.4111
3R3R	18 (35.3%)	17 (33.3%)		

*CI, confidence interval; VDR, Vitamin D Receptor; TYMS, Thymidylate Synthase*.

#### VDR *fok1* dominant allele model

Combining the Genotypes with the dominant mutant T allele also obtained a significant association with the ON finding. A dominant allele model was performed, and the results were as follows: The ON patients included 58.8% CT/TT with the T mutant allele, and 41.2% CC wild type. The patients who did not develop ON included 27.5 and 72.5% of the CT/TT and the CC polymorphism groups respectively. The positive correlation between the T allele and the ON had a *P*-value of 0.0025, and an odds ratio of 3.776, concluding that patients with the T allele are 3.8 times at higher risk to develop ON, as shown in Table 2.

#### TYMS tandem repeats and osteonecrosis

**Figure 5:**
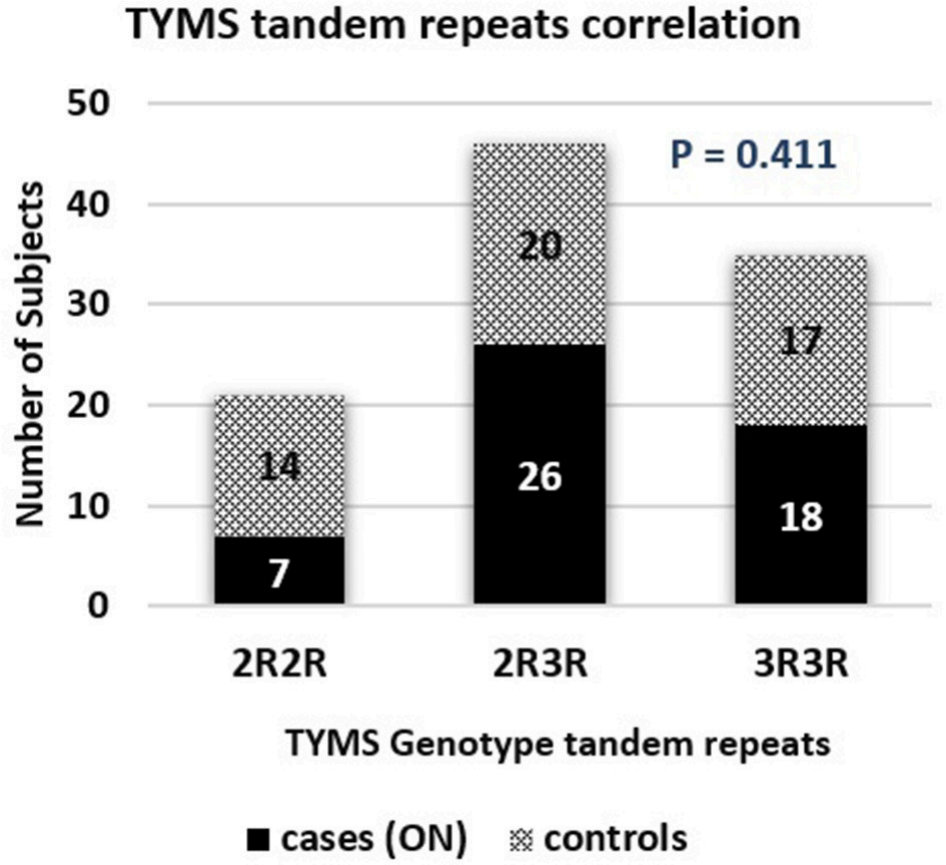
TYMS tandem repeats correlation bar chart in osteonecrosis cases and controls.

The TYMS tandem repeats were reported in the ON cases to be 13.7%, 51%, and 35.3% in the 2R2R, 2R3R, and 3R3R TYMS variation respectively. The control group revealed a distribution of 27.5% 2R2R, 39.2% 2R3R, and 33.3% for the 3R3R tandem repeat. According to the reported percentages the TYMS had no significant difference between the ON and Non-ON patients, with a *P*-value of 0.411 as shown in Table 2 and Figure 5.

## Discussion

Glucocorticoid-induced osteonecrosis is a complex multifactorial polygenic toxicity which results due to an interaction between a person's genetic makeup and various environmental factors (13). Generally, there are multiple risk factors for the incidence of GC induced ON such as age more than 10 years, female gender, and various controversial genetic polymorphisms (14).

Genetic polymorphisms in several genes including *SERPINE1, VDR, CYP3A4, TYMS, PAI 1, and ACP 1* were documented to be associated with osteonecrosis (2). A meta-analysis of genetic risk factors for glucocorticoid-induced osteonecrosis concluded that PAI-1 4G/5G and ABCB1- ATP Binding Cassette Subfamily B Member 1 polymorphisms are risk factors for osteonecrosis (15). However, these results were not replicated in later studies due to the intergenetic variability among different populations.

VDR gene polymorphisms were previously associated with osteopenia in non-cancer settings (16). Also, VDR is regarded as a regulator of cytochrome P450 3A4 expression and the p- glycoprotein excretion of glucocorticoids (17). The complex interdependent regulation of CYP3A, p-glycoprotein, and VDR, along with the effects of p-glycoprotein and CYP3A on the extent of drug absorption and metabolism, make a prior prediction of the functional consequences of genetic polymorphism for the VDR gene very difficult. The VDR gene polymorphisms (BsmI rs1544410, ApaI rs739837, and TaqI rs731236) have been previously studied; in association with Bone Mineral Density (BMD) levels among Egyptian Children with ALL by Tantawy et al. (18). In their study, the Tt genotype was correlated with high BMD as compared to other TaqI genotypes and had a positive significance with a *P*-value of 0.0420. There was a trend toward higher BMD with the genotype Bb as compared to other BsmI genotypes. Furthermore, no statistical significance was found between the other VDR genotypes or haplotypes studied and BMD (18). The VDR fok1 variant has been analyzed for its association with calcium absorption and BMD levels. Individuals with the TT mutant genotype had been reported to have less total calcium absorption, and less calcium deposit to the skeleton in both children and adults (19, 20).

VDR fok1 genotype was not previously studied in relation to glucocorticoid-induced osteonecrosis among the Egyptian population; neither in children nor in adults. However, it was investigated by St Jude hospital on the American children administering the same Total XV protocol as in our study (21). St Jude results revealed the association with the induced toxicity with a *P*-value of 0.045 and odds ratio of 45. Confirming the finding in this study, the VDR T mutant allele showed association with the incidence of glucocorticoid-induced osteonecrosis with a *P*-value of 0.0025.

Relling et al. investigated the role of other genetic polymorphisms that are associated with AVN in pediatric ALL. Among various SNPs, only polymorphisms in the VDR and TYMS were independent predictors for osteonecrosis (21). Also, other polymorphisms may contribute to the development of AVN, as polymorphisms in the folate pathway (e.g., methylenetetrahydrofolate reductase; (22) and polymorphisms in cytochrome P450 (23).

The other polymorphism selected in this exploratory analysis for genotyping was TYMS. The TYMS 2R2R genotype was associated with low TYMS expression in a previous study reported by Krajinovic et al.; rendering cells more susceptible to toxic and anticancer effects of TYMS inhibitors, which also included methotrexate (24). However, this finding could not be confirmed by subsequent genome-wide association studies (GWAS) performed by Karol et al. who reported no significance for the TYMS tandem repeats with the reported osteonecrosis (25).

Similarly, a report from Children's Oncology group found no association between TYMS and osteonecrosis with a *P*-value of 0.319 (26). In agreement with these results, the present study showed no link between TYMS tandem repeat variations and glucocorticoid-induced osteonecrosis among our Egyptian pediatric ALL patients with a *P*-value of 0.772.

The results of this study may have implications for modifying therapy decisions in the future. Dose adjustment of cancer therapeutics would potentially play a crucial role in optimizing treatment, due to the mild balance that exists between clinically effective and toxic drug exposures for most anticancer drugs. Pharmacokinetics and pharmacogenetics have been regarded as the most valuable tools that successfully guide dosing for many cancer therapies.

With glucocorticoids particularly dexamethasone, there are a few knowledge gaps that need to be filled before individualized dose application. There is currently no definite therapeutic window for dexamethasone proposed in ALL therapy. Therefore, planned prospective studies are required to further set the relationships between dexamethasone pharmacokinetics, clinical outcome, and toxicity in large patient populations, as this may facilitate the utility of drug exposure along with pharmacogenetics as a biomarker or predictive measure. Through the application of pharmacometrics and mathematical models, a personalized dosage of glucocorticoids could be made to serve the optimum efficacy with the least toxicity encountered during pediatric ALL treatment.

## Ethics statement

All guardians gave written informed consent in accordance with the Declaration of Helsinki. The protocol was approved by the Scientific and Medical Advisory Committee (SMAC).

## Author contributions

DE conducted the genotyping, performed the statistical analysis, interpreted the data, and drafted the manuscript. DY oversaw the genotyping data, drafted the manuscript, and supervised all laboratory methods performed. NA, SG, and IZ collected and analyzed all MRI data. HA contributed as a principal investigator to the project. IS designed the study, analyzed and interpreted the data, drafted the manuscript, and contributed as a principal investigator for the clinical protocol and the project.

### Conflict of interest statement

The authors declare that the research was conducted in the absence of any commercial or financial relationships that could be construed as a potential conflict of interest.
